# *MAOA* variants differ in oscillatory EEG & ECG activities in response to aggression-inducing stimuli

**DOI:** 10.1038/s41598-019-39103-7

**Published:** 2019-02-25

**Authors:** SeungYeong Im, Jinju Jeong, Gwonhyu Jin, Jiwoo Yeom, Janghwan Jekal, Sang-im Lee, Jung Ah Cho, Sukkyoo Lee, Youngmi Lee, Dae-Hwan Kim, Mijeong Bae, Jinhwa Heo, Cheil Moon, Chang-Hun Lee

**Affiliations:** 1 School of Undergraduate Studies, DGIST, Daegu, Korea; 2 Department of Brain and Cognitive Sciences, Graduate School, DGIST, Daegu, Korea; 3 Undergraduate School Administration Team, DGIST, Daegu, Korea; 4 Well Aging Research Center, DGIST, Daegu, Korea

**Keywords:** Personality, Aggression, Neurophysiology

## Abstract

Among the genetic variations in the monoamine oxidase A (*MAOA*) gene, upstream variable number tandem repeats (uVNTRs) of the promoter have been associated with individual differences in human physiology and aggressive behaviour. However, the evidence for a molecular or neural link between *MAOA* uVNTRs and aggression remains ambiguous. Additionally, the use of inconsistent promoter constructs in previous studies has added to the confusion. Therefore, it is necessary to demonstrate the genetic function of *MAOA* uVNTR and its effects on multiple aspects of aggression. Here, we identified three *MAOA* alleles in Koreans: the predominant 3.5R and 4.5R alleles, as well as the rare 2.5R allele. There was a minor difference in transcriptional efficiency between the 3.5R and 4.5R alleles, with the greatest value for the 2.5R allele, in contrast to existing research. Psychological indices of aggression did not differ among *MAOA* genotypes. However, our electroencephalogram and electrocardiogram results obtained under aggression-related stimulation revealed oscillatory changes as novel phenotypes that vary with the *MAOA* genotype. In particular, we observed prominent changes in frontal γ power and heart rate in 4.5R carriers of men. Our findings provide genetic insights into *MAOA* function and offer a neurobiological basis for various socio-emotional mechanisms in healthy individuals.

## Introduction

Aggression is a ubiquitous phenomenon that arises from anger or antipathy and often results in hostile or violent behaviour in humans. Pathological aggression related to antisociality or violence is highly heritable^1–5^, and previous work estimated that genetic factors account for approximately 50% of the variation in aggression^6^. Among candidate genes for aggression, mutations, deletions, single-nucleotide polymorphisms (SNPs), and variable number of tandem repeats (VNTRs) in the X-linked monoamine oxidase A (*MAOA*) gene were the first known genetic deficiencies in animal and human models^7–9^. The gene product monoamine oxidase A, which is expressed in specific cells of the human brain and peripheral tissues^10–12^, metabolizes monoamines, such as the neurotransmitters serotonin, norepinephrine, and dopamine, which are involved in the regulation of emotions^13–15^.

The 30-base pair (bp) upstream variable number tandem repeat (uVNTR) polymorphism of the *MAOA* promoter region is a major focus of studies on genetic associations with numerous phenotypes of aggression, such as antisocial behaviour^16^ and impulsivity^17^, as well as neuropsychiatric disorders including alcoholism^18,19^. *In vitro* gene fusion and transfection assays initially indicated that *MAOA* uVNTR variants may cause differential transcription of the *MAOA* mRNA; the 2-repeat (2R) and 3-repeat (3R) low-*MAOA* (L-*MAOA)* alleles are associated with lower transcriptional efficiency than the 4-repeat (4R) high-*MAOA* (H-*MAOA)* allele, with corresponding lower enzyme expression and higher predicted downstream neurotransmitter levels^20–23^. In addition, studies showing that the aggressive propensity of L-*MAOA* carriers becomes more apparent following traumatic experiences, such as childhood maltreatment, underscore the importance of gene × environment interactions in modulating the role of *MAOA* in aggression^16,24–26^. However, conflicting results suggest that the *MAOA* uVNTR alone or its interactions with the environment have no or opposing relationships with aggressive phenotypes^27–31^. In addition, studies have shown that *MAOA* uVNTR variants do not correspond to monoamine oxidase A levels in post-mortem brains with brain monoamine oxidase A activity in healthy men^32–34^, and the *in vitro* transcriptional efficiency of monoamine oxidase A was inconsistent and depended on the transfected constructs^35^. These findings suggest that factors such as post-translational factors or the modulation of protein levels and activity, and possibly compensatory mechanisms between the different neurotransmitter systems regulated by *MAOA*, may work together in modulating the role of *MAOA* uVNTR genotype on aggression.

While studies using positron emission tomography (PET) and [^11^C]clorgyline did not find an association between *MAOA* genotype and brain *MAOA* activity in healthy men^32,33^, studies using functional magnetic resonance imaging (fMRI) support an effect of the *MAOA* genotype on aggression-related functional changes in the nervous system. The *MAOA* genotype has been associated with the level or distribution of neural activity in cortical regions of the brain, such as the dorsal anterior cingulate cortex or the ventromedial prefrontal cortex, in response to Go/No-Go tasks or programmes including social exclusion^36,37^. In addition, heathy L-*MAOA* carriers showed prominent structural and functional changes in the corticolimbic network, which plays a crucial role in regulating emotional states during the recall of negative events^17^. Recent work showed that L-*MAOA* carriers are more susceptible to pharmacological regulation by serotonin, with changes in the corticolimbic network, indicating a possible link between the serotoninergic system and *MAOA* genotype^38^. The effects of *MAOA* genotype on the dopaminergic system remain controversial. For example, the dopamine levels in response to a violent video was not dependent on *MAOA* genotype^39^. As previously indicated, the conflicting results obtained by different studies could be due to the broad role of *MAOA* in modulating different neurotransmitter systems, the heterogeneity in measuring neural function and human behaviour, the *in vitro* system used to measure the effect of uVNTR on *MAOA* expression, and to the effect of gene × environmental interactions^40–43^. The results of such studies in women are also perplexing. It remains unclear whether *MAOA* is an X-inactivated gene, making it challenging to evaluate enzyme expression or activity in heterozygous genotypes^44^.

Beyond the conflicting results, historical evidence points towards an association of *MAOA* genotype to neural function in aggressive behaviour. Thus, in the present study, we investigated the effects of the *MAOA* genotype on multiple aspects of aggression, including neural and cardiac activities. In addition, given the fact that allelic distribution varies across ethnic/racial groups, we characterized the *MAOA* genotype distribution in the Korean population for the first time^22^. Transcriptional efficiency was assessed using *in vitro* reporter gene assays based on full *MAOA* promoter sequences to more accurately test the transcriptional contribution of the uVNTR. We also investigated the functional relationship between *MAOA* genotypes and aggression by analysing electroencephalogram (EEG)- and electrocardiogram (ECG)-based neurobiological responses to commonly encountered aggression stimuli in both men and women. In particular, EEG and ECG are useful tools for studying differences in neural activity because they are capable of measuring real-time responses and are highly accessible, with fine temporal resolution across the spectrum range of EEG and the direct autonomic reactivity range of ECG. Moreover, many EEG and ECG studies have shown that specific forms of oscillatory activity are associated with antisocial behaviour or aggressive tendencies^45–56^. In particular, frontal EEG asymmetry, which is a representative functional feature of aggression^45,46^, is affected by dopamine regulation^57^. Nevertheless, few studies of the relationship between *MAOA* genotypes and aggression have used EEG and ECG approaches in a pool of healthy adults.

## Results

### Genetic variation of *MAOA* in the Korean population

**Figure 1:**
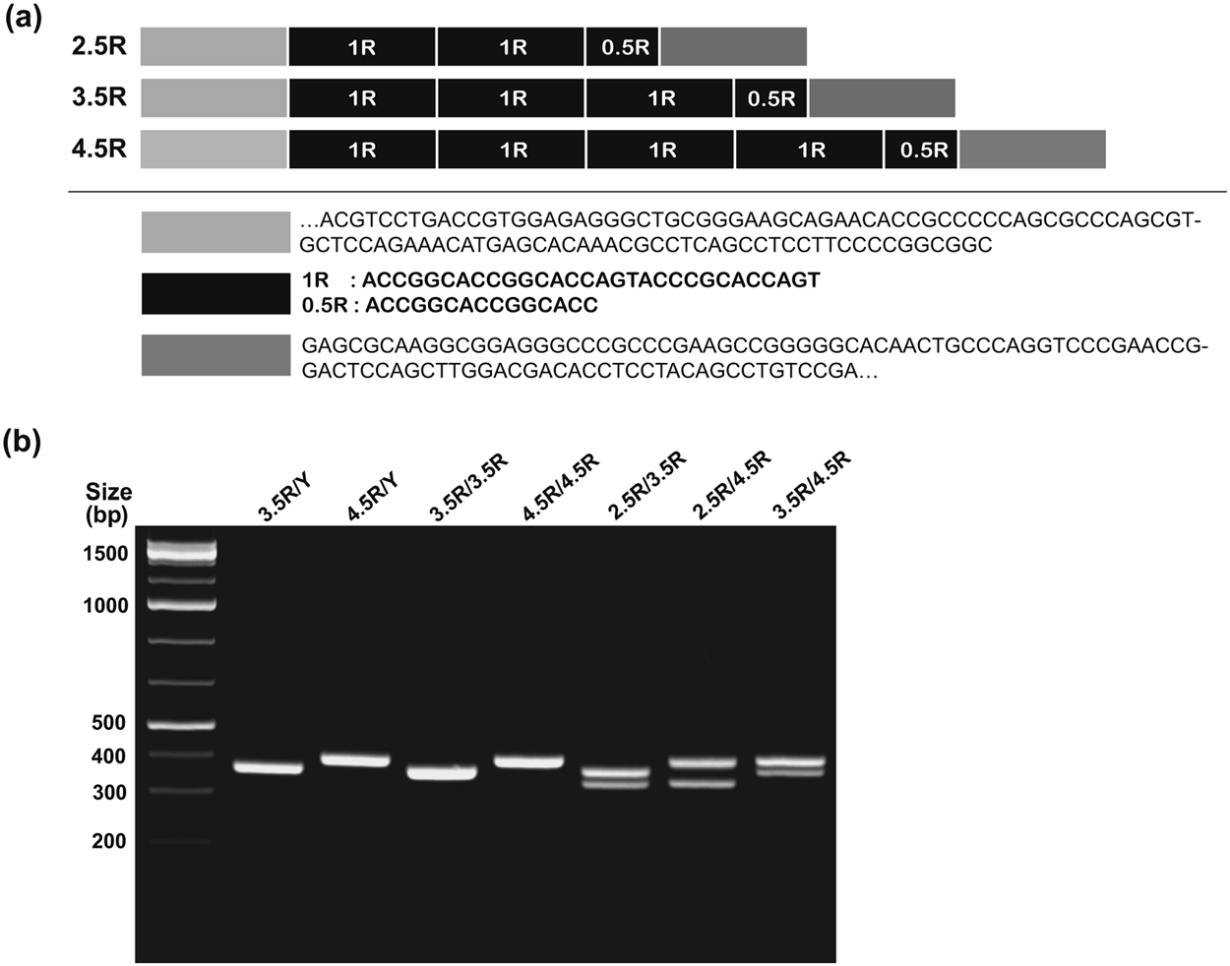
Genomic structure of the *MAOA* uVNTR polymorphism. (**a**) Nucleotide sequence alignment of the *MAOA* uVNTR alleles 2.5R, 3.5R, and 4.5R. 1R of each *MAOA* uVNTR allele consisted of a 30 bp “ACCGGCACCGGCACCAGTACCCGCACCAGT” sequence, followed by the first half-sequence of “ACCGGCACCGGCACC” in the last 0.5R. (**b**) Visualization of the different *MAOA* genotypes of the Korean population in 3% agarose gel. Lanes 1 and 2 are 3.5R/Y and 4.5R/Y from men; lanes 3, 4, 5, 6 and 7 are 3.5R/3.5R, 4.5R/4.5R, 2.5R/3.5R, 2.5R/4.5R, and 3.5R/4.5R from women, respectively. The full gel is presented in Fig. S7a.

To characterize genetic variation of *MAOA* in the Korean population, we identified *MAOA* uVNTR genotypes and allelic frequencies. Although many studies have followed the original method, which classifies alleles as 2R, 3R, 4R, and 5R^22,23,58^, some groups have suggested a new classification method for the sequence repeats, namely, 2.5R, 3.5R, 4.5R, and 5.5R^59–62^. The difference lies in the first 15 bp half-repeat sequence (−1141/−1127 bp), which is next to the repeated 30 bp sequence (−1262/−1142 bp)^63^; the redefined classification includes this sequence (Fig. 1a). Seven different *MAOA* genotypes were detected in our population, including two genotypes in men and five genotypes in women (Table 1). The polymerase chain reaction (PCR) products of the seven observed types of *MAOA* uVNTRs are shown in Figs 1b and S7a. Among the three *MAOA* uVNTR alleles detected, the 3.5R allele was the most prevalent (Table 1). The 2.5R allele appeared only in heterozygous genotypes, such as 2.5R/3.5R and 2.5R/4.5R in women, and at a very low frequency. The genotype distribution in our study was not in Hardy-Weinberg equilibrium (χ^2^ = 15.59, *df* = 4, χ^2^/df = 3.90, *p* (χ^2^ > 15.59) = 0.0036).

**Table 1:** Summary of *MAOA* genotypes and allelic frequencies in the Korean population.

Genotypes					Alleles		
		n	%		2.5R	3.5R	4.5R
**Men**	**Hemizygous**						
	**3.5**R**/Y**	178	68.5		0	178	0
	**4.5**R**/Y**	82	31.5		0	0	82
	**Total**	260	100		0	178	82
**Women**	**Homozygous**						
	**3.5**R**/3.5**R	106	40.2		0	212	0
	**4.5**R**/4.5**R	41	15.5		0	0	82
	**Heterozygous**						
	**2.5**R**/3.5**R	7	2.7		7	7	0
	**2.5**R**/4.5**R	4	1.5		4	0	4
	**3.5**R**/4.5**R	106	40.2		0	106	106
	**Total**	264	100		11	325	192
				**Total (n)**	11	503	274
				%	1.4	63.8	34.8

### Functional analysis of the promoter activity of *MAOA* uVNTR alleles

**Figure 2:**
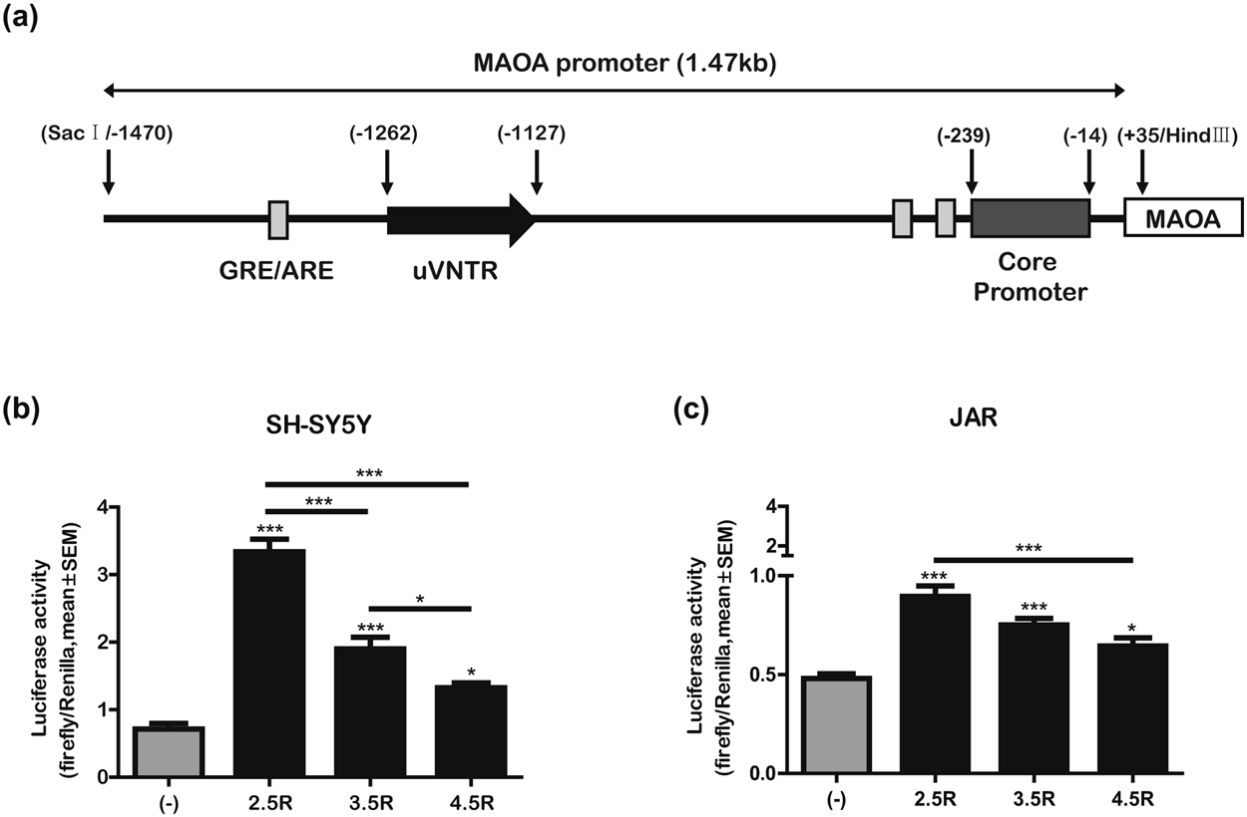
Transcriptional efficiency of *MAOA* promoter fusions. (**a**) Schematic diagram of the 1.5 kb promoter organization with the 4.5R allele. Sites from −1262 to −1127 are for the position of *MAOA* uVNTR alleles and sites −1470 to +35 are for cloning into the promoter fusion vector. The sites for the core promoter and three GRE/AREs (grey blocks) are also shown. GRE, Glucocorticoid response element; ARE, Androgen response element. (**b,c**) Reporter gene assays. *MAOA* promoter constructs carrying the 2.5R, 3.5R, or 4.5R alleles fused to firefly luciferase were co-transfected with a plasmid containing Renilla luciferase into SH-SY5Y cells and JAR cells. Empty pcDNA vector was used as a negative control (−). Data are presented as the ratio of firefly to Renilla luciferase activity (mean ± SEM) from one of three independent experiments with 4–8 wells. *, **, and *** above the horizontal lines indicate statistically significant differences among *MAOA* uVNTR alleles at *p* < 0.05, 0.01, and 0.001, respectively, by one-way ANOVA with Tukey’s post hoc test; * and ** above the bar graph indicate statistically significant differences between the negative control group and *MAOA* uVNTR alleles at *p* < 0.05 and 0.01, respectively, by one-way ANOVA with Tukey’s post hoc test.

To characterize *MAOA* uVNTR function *in vitro*, the transcriptional efficiency of the observed alleles was assessed in SH-SY5Y and JAR cells using reporter gene constructs containing the *MAOA* promoter region fused to the firefly luciferase gene. For each promoter construct, we used a full 1.47 kb upstream fragment from −1470 to +34 bp^63^, which encompasses three different *MAOA* uVNTR alleles (2.5R, 3.5R, and 4.5R) containing transcription-regulatory regions of *MAOA* (Fig. 2a). Reporter gene expression levels gradually decreased with each allele in the order of 2.5R, 3.5R, and 4.5R in both SH-SY5Y and JAR cells (Fig. 2b,c). Statistically significant differences were detected between the negative control condition and each allele (*F*, 65.41; *p* < 0.0001 for 2.5R; *p*, 0.0002 for 3.5R; and *p*, 0.0261 for 4.5R in SH-SY5Y cells; *F*, 20.14; *p* < 0.0001 for 2.5R; *p*, 0.0001 for 3.5R; *p*, 0.0239 for 4.5R in JAR cells). In a comparison among three alleles, 3.5R showed significantly higher *MAOA* transcriptional efficiency than 4.5R (*F*, 65.41, *p*, 0.0365), and the 2.5R allele showed the highest value, with statistical significance in SH-SY5Y cells (*F*, 65.41, *p* < 0.0001). There was also a significant difference in *MAOA* transcriptional efficiency between the 2.5R and 4.5R alleles in JAR cells (*F*, 20.14, *p*, 0.0003). Since the transcriptional efficiency decreased as the *MAOA* uVNTR sequence increased in length, we performed additional reporter gene assays with nine alleles spanning 2R-6R to determine the length-dependent effect of *MAOA* uVNTR on transcription. The PCR products of the promoters carrying each of the *MAOA* uVNTR allele-fused constructs, along with the corresponding observed *MAOA* genotypes, are shown (Figs S1a,b and S7b,c). Although the range of absolute values differed, the pattern of transcriptional efficiency for the 2.5R, 3.5R, and 4.5R alleles was preserved (*F*, 191.8; *p* < 0.0001 between 2.5R and 3.5R; *p* < 0.0001 between 2.5R and 4.5R; and *p* < 0.0351 between 3.5R and 4.5R in SH-SY5Y cells; *F*, 57.74; *p* < 0.0001 between 2.5R and 3.5R; *p*, 0.0345 between 2.5R and 4.5R; and *p*, ns between 3.5R and 4.5R in JAR cells); in addition, the 5.5R allele, which was found in other ethnic/racial groups^20,22^, showed a relatively higher value than those of the 3.5R and 4.5R alleles (Fig. S1c–f). However, no length-dependent effect of the *MAOA* uVNTR on transcription was observed. Nevertheless, our results showed higher transcriptional efficiency for the shorter uVNTR variants, 2.5R and 3.5R, that was more evident in SH-SY5Y cell line as compared to JAR. Due to the low frequency of the 2.5R allele in the population, we focused the next experiments on subject carrying the 3.5R and 4.5R alleles.

### Effects of *MAOA* genotypes on questionnaire scores

To test the relationship between *MAOA* genotypes and psychological aggression and to compare hemi- and homozygous 3.5R and 4.5R carriers, the scores of three self-report questionnaires (the Buss-Durkee Hostility Inventory (BDHI), the Buss-Perry Aggression Questionnaire (BPAQ), and the Peer Conflict Scale (PCS)) were analysed. No significant differences in the mean scores of either the 3.5R/Y and 4.5R/Y genotypes in men or the 3.5R/3.5R and 4.5R/4.5R genotypes in women were found on any of the questionnaires (Fig. S2). Similarly, no significant differences in the mean BPAQ and PCS subcategory scores were observed between genotypes. Therefore, the effect of the *MAOA* genotype on psychological aggression was negligible in our study population.

### Effects of *MAOA* genotypes on EEG response

**Figure 3:**
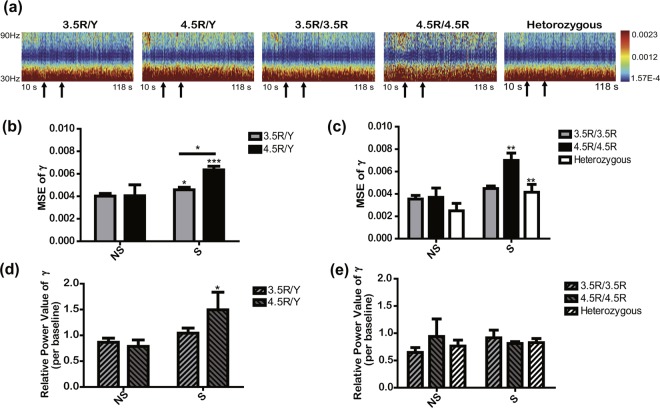
Comparisons of the EEG γ response at F8 across *MAOA* genotypes. (**a**) Power spectral density of the γ wave at F8. The panels represent power spectral density by time (10–117.75 s) and frequency (30–90 Hz) intervals across *MAOA* genotypes. At the bottom of each panel, the first arrow represents the start of the NS (19 s), and the second arrow represents the start of S (stimulus, 28 s). (**b,c**) represent the MSE value of the γ wave at F8 during NS and S between *MAOA* genotypes in men and women, respectively. (**d,e**) represent the relative power value of the γ wave at F8 during NS and S between *MAOA* genotypes in men and women, respectively. All values are represented as the means ± SEM. The sample size for EEG measurement was 84, including 36 men (21 for 3.5R/Y, 15 for 4.5R/Y) and 48 women (23 for 3.5R/3.5R, 5 for 4.5R/4.5R, and 20 for the heterozygous group). *, **, and *** above the horizontal lines indicate statistically significant differences between *MAOA* genotypes at *p* < 0.05, 0.01, and 0.001, respectively, by repeated measures of two-way ANOVA with Bonferroni post hoc test; * and ** above the graph bars indicate statistically significant differences between NS and S at *p* < 0.05 and 0.01, respectively, by repeated measures of two-way ANOVA with Bonferroni post hoc test.

We analysed EEG-based neurobiological responses to aggression-inducing stimuli to examine associations between *MAOA* allelic variation and neural or functional differences. When aggression-inducing stimuli were applied through the F8 channel, the power spectral density was displayed as a heat map according to the frequency interval (Fig. 3a). We assessed significant difference between the genotypes during S only for the EEG values with significant differences between NS and S in the analysis. Mean square error (MSE) analysis, which indicates the oscillatory variability of the time (neutral stimulus (NS) and stimulus (S)) and frequency (α, β, γ, θ, and δ waves) intervals, revealed that the MSE value of the γ wave in the F8 channel during S was significantly larger than that during NS in both men and women, except for the 3.5R/3.5R group (*t*, 2.425, *p*, 0.0416 for 3.5R/Y; *t*, 6.125, *p* < 0.0001 for 4.5R/Y; *t*, 3.692, *p*, 0.018 for 4.5R/4.5R; and *t*, 3.711, *p*, 0.0017 for heterozygous group) (Fig. 3b,c). Moreover, the MSE value of the γ wave in the F8 channel also differed significantly between *MAOA* genotypes during S in men; specifically, the MSE values of the 4.5R/Y group were larger than those of the 3.5R/Y group (*t*, 2.375, *p*, 0.0408). However, for the relative power value of the γ wave in the F8 channel during S, the values did not differ significantly by *MAOA* genotype both in men and women (Fig. 3d,e). In addition, for the other four frequency intervals in the F8 channel and the five frequency intervals in the Fp2 channel, the MSE values during S did not show significant differences between genotypes (Figs. S3 and S4). For the relative power value of the five frequency intervals in the Fp2 and F8 channels, significant differences between genotypes were not observed as well (Figs. S3 and S4). Therefore, when aggression-inducing stimuli were applied, the oscillatory change in the γ wave in the F8 channel was greater in hemizygous 4.5R carriers than in hemizygous 3.5R carriers.

### Effects of *MAOA* genotype on ECG responses

**Figure 4:**
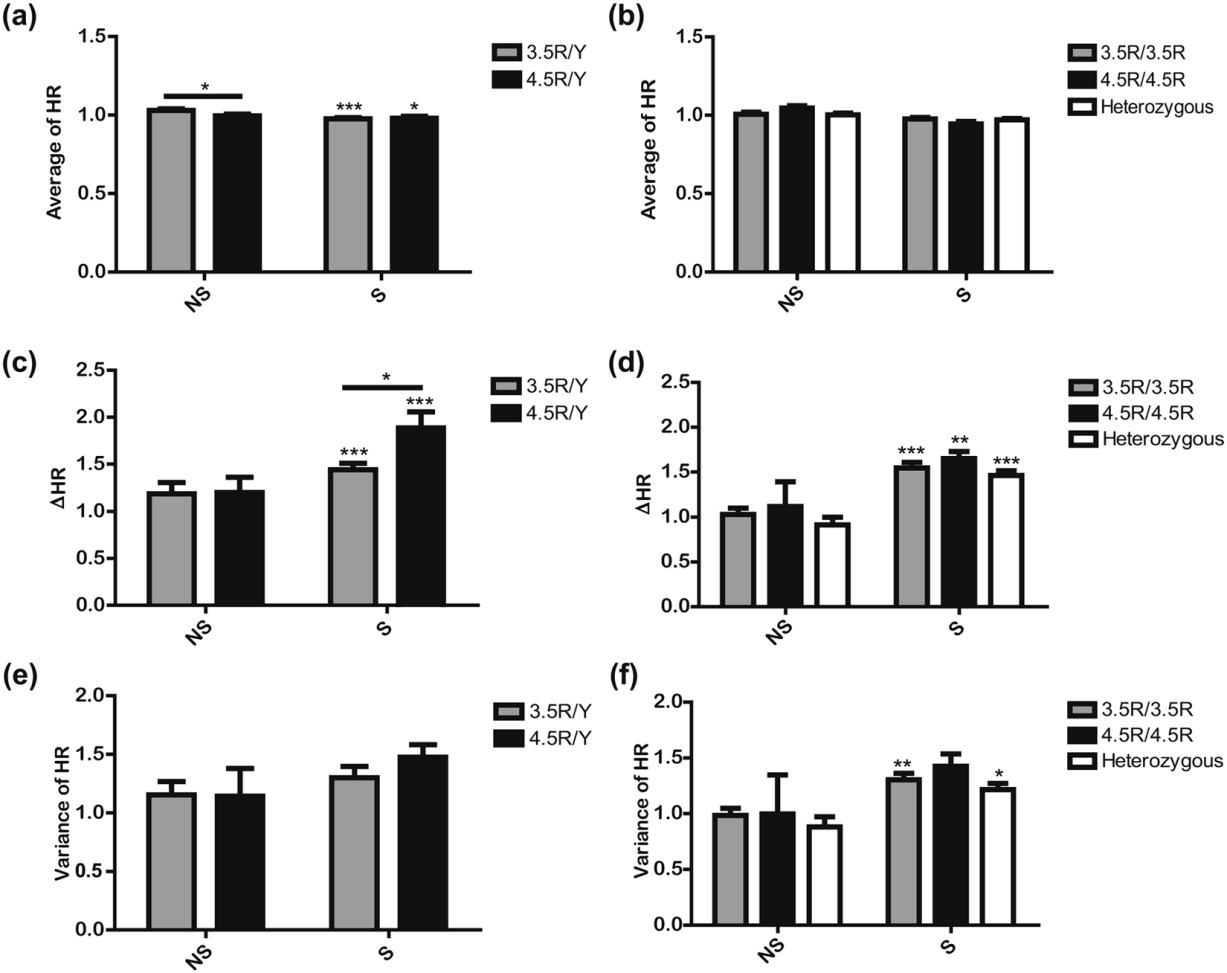
Comparison of ECG across *MAOA* genotypes. ECG signals were used to calculate HR (unit: BPM) and were converted to values relative to baseline. (**a,b**) represent average HR during NS and S between *MAOA* genotypes in men and women, respectively. (**c,d**) represent ΔHR during NS and S between *MAOA* genotypes in men and women, respectively. (**e,f**) represent variance in HR during NS and S between *MAOA* genotypes in men and women, respectively. All values are represented as the means ± SEM. The ECG sample size was 70, including 30 men (18 for 3.5R/Y, 12 for 4.5R/Y) and 40 women, (20 for 3.5R/3.5R, 3 for 4.5R/4.5R, and 17 for the heterozygous group). * and ** above the horizontal line indicate statistically significant differences between *MAOA* genotypes at *p* < 0.05 and 0.01, respectively, by repeated measures of two-way ANOVA with Bonferroni post hoc test; *, **, and *** above the graph bar indicate statistically significant differences between NS and S at *p* < 0.05, 0.01, and 0.001, respectively, by repeated measures of two-way ANOVA with Bonferroni post hoc test.

We analysed the heart rates (HRs) from the ECG results to investigate autonomic reactivity related to aggression according to the *MAOA* genotype. Since low HR in the resting state is a marker for aggression^48,64–67^, we assessed significant differences between the genotypes during NS only for the average HR values, with significant differences between NS and S in the analysis. The average HR was significantly lower for 4.5R/Y than for 3.5R/Y during NS (*t*, 2.604, *p*, 0.0234), and the values were significantly higher in both genotypes during NS than during S in men (Fig. 4a). However, the average HR was not significantly different either between genotypes or between scenes (NS vs. S) in women (Fig. 4b).

Since ΔHR and HR variance indicate the degree of change in autonomic reactivity, we assessed significant differences between the genotypes during S only for the values, with significant differences between NS and S in the analysis. ΔHR was significantly higher during S than during NS in all genotypes (*t*, 4.975 *p* < 0.0001 for 3.5R/Y; *t*, 6.929 *p* < 0.0001 for 4.5R/Y; *t*, 8.333, *p* < 0.0001 for 3.5R/3.5R; *t*, 3.716, *p*, 0.0020 for 4.5R/4.5R; and *t*, 7.537, *p* < 0.0001 in the heterozygous group) (Fig. 4c,d). In particular, ΔHR was significantly higher in the 4.5R/Y group than in the 3.5R/Y group during S in men (*t*, 2.364, *p*, 0.0426) (Fig. 4c). However, no such difference in ΔHR between genotypes was observed in women (Fig. 4d). In addition, the variance of HR was not significantly different either between genotypes or between scenes both in men and women (Fig. 4e,f). Taken together, the ΔHR by aggression-inducing stimuli was greater in hemizygous 4.5R carriers than in hemizygous 3.5R carriers, while average HR by neutral stimuli was lower in hemizygous 4.5R carriers than in hemizygous 3.5R carriers in men.

## Discussion

*MAOA* has long been a gene of particular interest for studying the association between genetic variation and behavioural or neurobiological characteristics associated with aggression^16,17,24–26,36–38,68^. However, despite numerous publications on this matter, studies are still needed to investigate whether some relationships are present in all racial groups^69^. Our research found that the frequencies of the common 3.5R and 4.5R alleles in Koreans, 63.8% and 34.8%, respectively, differed from the observed frequencies of 35–39% and 59–63% for 3R and 4R, respectively, in Caucasians (assumed to be equivalent to 3.5R and 4.5R in our classification)^22,60,70^. The allele frequency observed here was similar to those previously reported for Chinese (54.5–64.7% and 34.8–44.2% for 3R and 4R, respectively)^35,69,71^ and Japanese (62% and 38% for 3R and 4R, respectively)^72^ individuals. Although not based on sequence analyses, these data provide a basis for the representative genetic distribution of *MAOA* uVNTR in Asians. We verified the allele frequency difference in *MAOA* uVNTR by race and geographic location^22,70,73,74^. Previous studies showed that 2R was rare in Asians^35,71,72^ and that 5R was found only in Caucasians^22,60^. In our study, we observed 2.5R at a very low frequency, which corresponds to 2R in previous studies.

The molecular basis of the difference in aggression between *MAOA* genotypes has largely been pursued in terms of expression levels using *in vitro* systems. These studies began with Sabol’s initial finding *in vitro*, which provided evidence for classifying *MAOA* genotypes as H-*MAOA* and L-*MAOA*^22^. The results for the most frequent alleles, which demonstrated lower promoter activities for 3R than for 4R, were replicated by others^20,23^, but these studies also showed inconsistent results for the relative promoter activity of the 5R allele, which were not noted due to its rare frequency^20,22^. We found that the differences in promoter activity among the three alleles were inconsistent with previous findings^20,22,23^; in our results, the difference between the 3.5R and 4.5R alleles was minor and dependent on the cell line used, with the 2.5R allele showing the highest transcriptional efficiency. Although our results are consistent with one previous report, this result was not discussed further due to the use of glioblastoma cell lines rather than neuroblastoma^35^. These discrepancies could be attributed to the different *MAOA*-luciferase reporter gene constructs used and to the length of the promoter studied. Most studies include the *MAOA* promoter fragment up to 1.3 kb upstream of the transcription start site, including the core transcriptional regulatory regions of *MAOA*, such as the four known Sp1 sites (−239/−14 bp), the SRY-binding site (−117/−111 bp), and the GR/AR response element (−289/−275 bp)^63,75–79^. However, it remains unclear whether an upstream region beyond 1.3 kb affects the transcriptional efficiency of *MAOA* uVNTR alleles; in particular, the results obtained using extended sequences (−1470/+34 bp, in our study; −1396/0 bp^35^) reached conclusions that differed from those of other studies (−1308/−6 bp^22^; −1336/−64 bp^23^; −1370/−7 bp^20^) in terms of the difference between the 3R and 4R alleles. Similar results have been reported regarding the 5R allele (−1308/−6 bp^22^ vs. −1370/−7 bp^20^; −1470/+34 bp, in our study; –1396/0 bp^35^). Additionally, the GR/AR response element (−1350/−1335 bp) and its upstream region in the *MAOA* promoter affect basal luciferase activity but do not respond to glucocorticoids or androgens^78^. Our results show that, using the full promoter under *in vitro* conditions, the promoter activity is higher in shorter uVNTR variants, although there is no a length dependent effect for variants ranging from 2.5R up to 6R. This finding suggests that the *MAOA* uVNTR may not be the only cis-regulatory element modulating *MAOA* transcriptional efficiency. For instance, epigenetic modification of the genome, such as DNA methylation, can be proposed as a potential mechanism that contributes to *MAOA* expression^32,33^. Indeed, *MAOA* promoter methylation in white blood cells has been associated with brain *MAOA* activity measured by PET. Future studies of *MAOA* promoter methylation and transcriptional efficiency will provide more reliable results and mechanisms related to the role of the *MAOA* uVNTR in transcription.

Beyond the ongoing studies to decipher the molecular mechanisms modulating *MAOA* levels, many studies have demonstrated functional phenotypes associated with H-*MAOA* and L-*MAOA* carriers^17,36–38^. We also discovered that EEG and ECG responses related to aggression differed with respect to the *MAOA* genotype. We found that hemizygous 4.5R carriers showed greater oscillatory variability in the right frontal γ wave in response to aggression-inducing stimuli. The EEG signal in the frontal region has mainly been studied with respect to the asymmetrical activity pattern of the α wave, which is related to specific types of aggression, such as positive or negative affect^45,46,49^. However, no effects were observed for the α wave of the right hemisphere in our results. Therefore, we can raise the possibility of other candidates. In early investigation, enhanced cortical δ wave activity was associated with violence or antisociality^50,80^. Studies of the γ wave in relation to aggression are relatively rare, but some reports suggested that the frontal γ wave shows increased activity following unpleasant stimuli^81^ and is also involved in impulse control in relation to addiction^82^. Thus, the greater γ responses of 4.5R carriers in our results might be associated with impulsive tendencies. Even though many neuroimaging studies have also shown that neural activity in the prefrontal/frontal cortex or corticolimbic neural circuits are related to aggressive behaviours, such as impulsivity^83–86^, this evidence is insufficient to causally link individual brain areas to specific EEG frequencies, including the γ wave. Moreover, variations in the γ wave might be induced during visual processing. Growing evidence indicates that this oscillatory variation can be driven by micro-saccadic movement or cross-frequency coupling of θ waves caused by this movement^87,88^. First, transient increases in γ power due to micro-saccadic movement generally occur in the 200–300 ms range^88^. However, the latency and duration of this effect have differed among some MEG studies^89–91^. Second, there is neural communication related to induced γ oscillation in visual cortical areas^87^. However, whether γ synchronization occurs between the visual cortex and the prefrontal/frontal cortex is unclear. Future research on γ dynamics caused by visual cues should be supplemented with eye-tracking systems or correction methods, such as averaging trials. Taken together, our results suggest that the level of oscillatory change in the frontal γ wave under aggression-inducing stimuli can be another functional phenotype that varies with the *MAOA* genotype in relation to aggression.

Furthermore, we discovered differences in ECG responses to aggression-inducing stimuli between *MAOA* genotypes. Previous studies proposed that a low HR during resting state is among the best-replicated physiological characteristics of aggression^48,64–67^, which may also indicate a fearless temperament^92^. We found a lower average HR under NS in hemizygous 4.5R carriers than in 3.5R carriers in men. Therefore, the physiological phenotypes of HR in 4.5R carrier will often be closely interrelated aggressive tendencies. Meanwhile, in our results, the average HR decreased in response to stimuli in men. Comparisons of average HR in response to emotional stimuli in adults with more and less aggressive tendencies or with and without a history of violent behaviour have revealed inconsistencies across studies^65,80,93,94^. Since HR variabilities are regulated by interactions between the vagal (which reduces HR) and sympathetic (which increases HR) systems^95^, these values have been used to measure emotional regulation capacity during psychological processes^80,96,97^. Then we remain focused on interpreting HR variabilities rather than average HR in the analyses under aggression stimulation. The correlation between HR variabilities and antisocial behaviour is not yet clear^98^; the possible connection between 4.5R carriers with larger HR variabilities under aggression stimuli and higher aggression is open to debate. Nevertheless, the different HR variability dynamics in *MAOA*-deficient mice support the feasibility of differences in HR variabilities in humans according to *MAOA* uVNTR genotype^99^. Therefore, this result implies that HR variabilities in response to aggression stimuli may be a neurobiological phenotype associated with genetic variations in *MAOA*. Furthermore, the use of human cardiomyocytes which contain *MAOA*^100,101^ has potential for studying enzyme expression and activity in the heart according to *MAOA* genotype indirectly. Although *MAOA* studies in human cardiomyocytes have still focused on the role of myocardial regulation of ROS^102,103^, there is evidence that *MAOA* is involved in the catabolism of norepinephrine in mouse cardiomyocytes^104^. This system can be suitable for predicting intracellular mechanism of *MAOA* in heart which corresponds to the phenotypes of HR between *MAOA* genotypes.

As the *MAOA* gene can be expected to follow the principles of X-linked gene expression, research in women is likely to have multiple outcomes with regard to aggression. Our EEG and ECG results in women with homozygous *MAOA* genotypes were inconsistent with those in men, as well as statistical significance was not obtained for women because of the low number of homozygous 4.5R carriers. Moreover, our findings showed no significant neurobiological response in women with heterozygous *MAOA* genotypes, similar to other studies^105,106^. This difference highlights the need for research at the cellular level regarding whether X-inactivation occurs in heterozygous *MAOA* genotypes to better understand genetic effects on aggression in women.

In this study, we identified new functional phenotypes of oscillatory EEG and ECG activities induced by aggression-related stimuli that vary with *MAOA* genotypes: greater reactivity of the γ signals and greater HR variabilities in 4.5R carriers than in 3.5R carriers. This result suggests that *MAOA* does have some genetic effects on neural mechanisms and may extend to emotional control mechanisms related to aggression. We also attempted to provide a basis for the neural mechanism indirectly through transcriptional expression according to *MAOA* uVNTR alleles *via* a reporter gene assay *in vitro*. Our study calls for further investigation into the contribution of uVNTR and additional mechanisms to *MAOA* transcription. Future studies should address two points. First, we must clarify whether *MAOA* uVNTR modulates the expression and activity of the enzyme, as well as neurotransmitter levels, neural activity and behaviour. Second, further studies are required to understand the differences in oscillatory activity from cognitive and emotional perspectives. Addressing these points will facilitate the interpretation of various human socio-emotional behaviours, such as depression^107^, anxiety^108^, and prosociality^109^, as well as cognitive abilities such as memory^110,111^, with regards to genetic variations in *MAOA*.

## Methods

### Study subjects

In the present study, 524 college and high school students (260 men and 264 women, age: 18.0 ± 1.4) in Korea were recruited and provided oral epithelial cells for genotyping. All subjects had no record of pathological behaviour and no criminal record. Of the total sample, 334 participants (169 men and 165 women) completed three self-report questionnaires. Of the participants who completed the questionnaires, 94 participants were randomly selected, and their neurobiological signals were measured. Since, the difference in brain wiring between right-handed and left-handed individuals remains unclear^112–115^, to avoid bias, we decided to select only right-handed individuals as our subjects. We collected valid data from 84 participants (36 men and 48 women) using EEG measurements and from 70 participants (30 men and 40 women) using ECG measurements. EEG samples with poor electrode attachment or subject movement were excluded from the analysis. ECG samples that were not consistent with PQRST waveforms, i.e., the series of the electrical events of heart rhythms (i.e., the P wave represents atrial depolarization, the QRS complex represents ventricular depolarization and contraction, and the T wave represents repolarization of ventricles)^116^ were also excluded from the analysis. This study was approved by the Institutional Review Board (IRB) of DGIST [Number: DGIST_170614-HR-009-04]. All participants provided written informed consent after hearing a detailed explanation of the experimental procedures, in accordance with the Declaration of Helsinki.

### Identification of genotypes

Genomic DNA was extracted from epithelial cells using the G-spin™ Total DNA Extraction Kit (7046, BOCA Scientific, USA). Polymerase chain reaction (PCR) fragments were amplified using the following primers: *MAOA* forward (−1386, 5′-GCTGGTCTCTAAGAGTGGGTAC-3′, −1366) and *MAOA* reverse (−1019, 5′-GAACGGACGCTCCATTCGGAC-3′, −1038)^35,63^. For *MAOA* genotyping, PCR was performed in 50 μL reactions containing 1 μL of each primer (10 pmol/μL), approximately 100 ng of genomic DNA, and 2X TaKaRa Ex Taq PCR Premix (RR001A, Takara, Korea). Amplification was performed under the following conditions: 34 cycles of 30 s denaturing at 95 °C and 120 s annealing and elongation at 68 °C, in accordance with the manufacturer’s instructions. In addition, all PCR products were purified using a PureLink™ Quick Gel Extraction and PCR Purification Combo Kit (Invitrogen, USA). The purified amplicons were split into two aliquots; one was submitted to an automated Sanger’ sequencing service (Cosmogenetech, Korea), and the other was separated *via* 3% agarose gel electrophoresis and visualized to confirm all genotypes twice, especially for heterozygous genotypes. The alleles of the *MAOA* uVNTR-repeated region were genetically identified based on a redefined genetic characterization method that include copies of the 1R sequence and the first half of 0.5R^22,59–62^. We followed the naming convention of 2.5R, 3.5R, and 4.5R in this study, which is assumed to be equivalent to 2R, 3R, and 4R in other studies^22,23,58^.

### Construct generation

DNA fragments containing the 2R, 2.5R, 3R, 3.5R, 4R, 4.5R, 5R, 5.5R, and 6R alleles of the *MAOA* gene promoter were generated by DNA synthesis (Cosmogenetech, Korea) based on the *MAOA* DNA sequence^63^ (GenBank accession number m89636). The alleles were PCR-amplified using two primers containing SacI (−1470, 5′-ACTGGCCGGTACCTGAGCTCCTGCAGCGAGCG-3′) and HindIII (+35, 5′-CCGGATTGCCAAGCTTGCTTTGGCTGAC-3′) restriction sites. The 1442–1577 bp DNA fragments containing the *MAOA* gene promoter were digested with restriction enzymes and ligated into the pGL4.10 [luc] vector (Promega, USA). All constructs were verified by sequencing (Cosmogenetech, Korea).

### Cell lines, transfection, and reporter gene assays

We used the brain-derived SH-SY5Y human neuroblastoma cell line and the JAR human placental choriocarcinoma cell line that have been previously used to the study the *in vitro* functional characteristics of *MAOA*^22^. SH-SY5Y cells exhibit prominent features of catecholaminergic neurons, in which *MAOA* is predominantly found^117^, and are used as an *in vitro* model of the dopaminergic system, which expresses tyrosine hydroxylase and dopamine-beta-hydroxylase, as well as the dopamine transporter^118,119^. SH-SY5Y cells are also driven towards adrenergic phenotypes under certain growth conditions^120,121^. JAR cells are derived from placental trophoblasts, in which *MAOA* is expressed^122,123^, and are used to study the serotonergic system, especially the serotonin transporter^124,125^. Firefly DNA and control TK DNA (pGL4.74, Promega, USA) were co-transfected into SH-SY5Y (ATCC, CRL2266) and JAR (ATCC, HTB144) cells using the SF Cell Line 4D-Nucleofector® X Kit (Lonza, Switzerland) using a 4D-Nucleofector system (Lonza, Switzerland) according to the manufacturer’s instructions. pcDNA and control TK DNA were co-transfected as negative controls. For each transfection, 2 × 10^6^ cells were resuspended in 100 µL of *Nucleofector* solution, 1 µg of firefly DNA and 1 µg of control DNA was added in a single Nucleocuvette^TM^, and the electroporation settings recommended for the cell line by 4D-Nucleofector system were applied (Pulse code: CM137). Transfected cells were plated at a density of 1 × 10^4^ cells/well in 96-well plates with culture medium and were cultured at 37 °C and 5% CO_2_ for 48 h and then lysed with 50 µL of PLB solution in each well following the instructions of the Dual-Luciferase Reporter Assay System (Promega, USA). Next, 100 µL of LAR II solution was added to 20 µL of transferred PLB lysate in a luminometer plate, and firefly luciferase activity was measured. After reading, 100 µL of STOP & Glo reagent was added, and Renilla luciferase activity was measured. Luminescence was measured on a Spark 10 M (Tecan, Switzerland), and the results are shown as the ratio of firefly luciferase to Renilla luciferase. Tests were repeated in 6–12 wells using the transfected cells, and two or three independent experiments were performed.

### Self-report questionnaires

The following three self-report questionnaires on aggression were used in this study: K-BDHI^126^, adapted from the Buss-Durkee Hostility Inventory (BDHI)^127^; K-BPAQ^128^, adapted from the Buss-Perry Aggression Questionnaire (BPAQ)^129^, which includes plan failure scenarios^130,131^; and K-PCS^132^, adapted from the Peer Conflict Scale (PCS)^133^. The peer-to-peer conflict scales for adolescents were classified into four factors: overt/relational and proactive/reactive. All questionnaires were validated in the Korean population^134–136^.

### Stimuli and procedure

Experiments were conducted in a soundproof room maintained at 25 °C and constant humidity, with one subject and one experimenter in pairs. Neurobiological signals were measured while the subject watched a 118-s-long video that consisted of ‘fixation cross (4 s) - black - neutral scene (5 s) - black - scene 1 (39 s) - black - scene 2 (32 s) - black - scene 3 (11 s) - end’ (Fig. S5). We used a fixation cross set against a white background, as well as four black scenes between the other scenes, as the baseline. We used a scene of an empty school classroom, which is a common sight in the daily lives of the subjects, as the neutral stimulus (NS). Three scenes (scene 1, scene 2, and scene 3) related to aggression were used as the stimulus (S). Scene 1 showed an abusive argument between a driver and bossy passenger on a bus, as verbal abuse is reported to be related to verbal aggression^137^. Scene 2 showed a chat screen of peer conflict, in which one student is bullied and burdened with all of the assignment. Bullying has been reported to trigger proactive and reactive aggression in adolescents^138,139^. Scene 3 showed a person scratching a blackboard with their nails, which has been reported to lead to irritability^140,141^.

### Data acquisition, processing, and analysis

Neurobiological signals were measured using an MP36 (BIOPAC, CA, USA) with a 4-channel system (sampling rate = 1000 Hz, bandpass filter = 0.5–100 Hz), which is useful for quick and simple measurements in large populations (sampling rate = 1000 Hz, bandpass filter = 0.5–100 Hz). In studies analysing the neurobiological responses of aggression using EEG, frontal α asymmetry is a well-known signal pattern for aggression^45,46^. Reportedly, relatively greater left frontal α asymmetry at resting state is an indicator of approach motivation, while greater right asymmetry is an indicator of avoidance motivation^45,142^. Similar results have also been obtained for the β signal^49^. Our subjects, Koreans, tend to show indirect emotional expressions such as avoidance of irritability compared with Westerners in several cross-cultural studies^143^. Additionally, studies of EEG responses to neutral, happy, and fear-related sounds in adult Korean men showed differences in α, β, and γ power values between emotional stimuli, especially in the right frontal region (Fp2 and F8 channels), compared with the left frontal region (F3, Fp1, F7)^144^. Therefore, we decided to analyse individual differences in the EEG signals concentrated on the right frontal region, Fp2 and F8, rather than analysing the asymmetric difference between the left and right frontal regions. Moreover, in control experiments, Fp2 and F8 showed prominent responses to the stimuli, while the differences in responses from P7 and P8 on stimulation were insignificant (Fig. S6). These two channels are not only accessible but are also related to aggression^46^. In our study, EEG was recorded through two electrodes on Fp2 (right side of prefrontal cortex) and F8 (right side of dorsolateral prefrontal cortex), with ground and reference electrodes on the neck, in accordance with the BIOPAC EEG electrode placement guidelines and the International 10–20 EEG system^145–147^. For EOG (Electrooculogram), an active electrode was placed above the left eye, with a ground electrode on the mid-forehead and a reference electrode on the left cheek bone for removal of artefacts from eye-movement or gross muscle movement. These artefacts were removed by independent component analysis using BIOPAC AcqKnowledge program version 4.2. For ECG, an active electrode was place on the right wrist, with a ground electrode on the right foot and a reference electrode on the left wrist, following Einthoven’s triangle. For data analysis, we targeted all frequency intervals, as many studies have shown that α, β, γ, and θ signals in the frontal regions are related to aggression^45,46,49,82,148^. For the signals from the Fp2 and F8 channels of each subject, discrete Fourier transforms were performed using the fast Fourier transform algorithm in MATLAB. Power spectral density was calculated in the area of each frequency interval (0.5 and 4 (δ), 4 and 8 (θ), 8 and 13 (α), 13 and 30 (β), and 30 and 90 (γ)) by the trapezoidal method. The mean value of the power spectral density of each frequency interval during neutral stimulus or stimulus was normalized to the mean value at baseline and was then expressed as the relative power value. We performed a 2000-point fast Fourier transform by dividing the data into 0.25 s intervals to trace dynamic changes in EEG over time. The values from each participant were normalized to area of the whole frequency domain (0–90 Hz). The mean values from all participants were then visualized by heat-map analysis. Apart from mean values, we calculated the power spectral density of each participant^149^. The average squared difference between the power spectral density for all 0.25 s intervals and measured values for each 0.25 s interval during NS or S at frequency intervals were presented as MSE. The ECG signal was corrected by detrending using the method of least squares, and R-R intervals (unit: ms) were obtained by calculating the local maximum value per heartbeat period. These were converted to HR values (unit: BPM) by 60000/(R-R intervals)^150^. The standard deviation of HR was calculated as the variance of HR, and the difference between the maximum and minimum values was calculated as ΔHR. The level of HR variabilities was then analysed using the variance of HR and ΔHR.

$${\rm{MSE}}=\{\sum {(AVG-value)}^{2}\}/n$$

### Statistical analysis

All statistical analyses were performed using Prism 8.0. One-way ANOVA with Tukey’s post hoc test was used to identify statistically significant differences in transcriptional efficiency values between allelic groups or between allelic groups and the negative control group. Since *MAOA* is located on the X chromosome, the results from men and women were analysed separately. To identify significant differences in the scores of questionnaires and their subcategories between genotypes, we used two-way ANOVA with the Bonferroni post hoc test. EEG results were analysed by separating the five frequency intervals (α, β, γ, θ, and δ). Repeated measures of two-way ANOVA with the Bonferroni post hoc test was used to determine statistically significant differences in EEG and ECG values between NS and S for each genotype. To determine significant differences in EEG and ECG values between genotypes during NS or S, we also used repeated measures of two-way ANOVA with the Bonferroni post hoc test. Statistical significance was set at *p* < 0.05.

## Supplementary information

Supplementary information
