# Publisher Correction: *MAOA* variants differ in oscillatory EEG & ECG activities in response to aggression-inducing stimuli

**DOI:** 10.1038/s41598-019-45227-7

**Published:** 2019-06-26

**Authors:** SeungYeong Im, Jinju Jeong, Gwonhyu Jin, Jiwoo Yeom, Janghwan Jekal, Sang-im Lee, Jung Ah Cho, Sukkyoo Lee, Youngmi Lee, Dae-Hwan Kim, Mijeong Bae, Jinhwa Heo, Cheil Moon, Chang-Hun Lee

**Affiliations:** 1 School of Undergraduate Studies, DGIST, Daegu, Korea; 2 Department of Brain and Cognitive Sciences, Graduate School, DGIST, Daegu, Korea; 3 Undergraduate School Administration Team, DGIST, Daegu, Korea; 4 Well Aging Research Center, DGIST, Daegu, Korea

Correction to: *Scientific Reports* 10.1038/s41598-019-39103-7, published online 25 February 2019

The original version of this Article contained a typographical error in the spelling of the author SeungYeong Im, which was incorrectly given as Seung Yeong Im. This has now been corrected in the PDF and HTML versions of the Article.

