# Supplementary material for: MAOA variants differ in oscillatory EEG & ECG activities in response to aggression-inducing stimuli
Source: Sci Rep. 2019 Feb 25;9:2680. doi: 10.1038/s41598-019-39103-7 (PMC6390082; doi:10.1038/s41598-019-39103-7)

**Title:** *MAOA* variants differ in oscillatory EEG & ECG activities in response to aggression-inducing stimuli

**Short title:** *MAOA* variants affect EEG & ECG activity

SeungYeong Im^a,b,†^, Jinju Jeong^c,d,†^, Gwonhyu Jin^a,‡^, Jiwoo Yeom^a,‡^, Janghwan Jekal^a,‡^, Sang-im Lee^a,‡^, Jung Ah Cho^a^, Sukkyoo Lee^a^, Youngmi Lee^a^, Dae-Hwan Kim^a^, Mijeong Bae^a^, Jinhwa Heo^a^ and Cheil Moon^b,*^ & Chang-Hun Lee^a,*^

^a^School of Undergraduate Studies, DGIST, Daegu, Korea

^b^Department of Brain and Cognitive Sciences, Graduate School, DGIST, Daegu, Korea

^c^Undergraduate School Administration Team, DGIST, Daegu, Korea

^d^Well Aging Research Center, DGIST, Daegu, Korea

†These authors contributed equally to this work.

^‡^These authors also contributed equally to this work.

^*^To whom correspondence should be addressed. E-mail: [leech@dgist.ac.kr](mailto:leech@dgist.ac.kr), cmoon@dgist.ac.kr

**Supplementary Information**

**Supplementary Figure 1. Transcriptional efficiency of *MAOA* promoter fusions from 2R to 6R.**

(a) Observed 2-6R alleles from the promoter carrying each of the *MAOA* uVNTR-fused constructs on 8% urea PAGE. (b) Comparison of observed genotypes of *MAOA* uVNTR and synthesized constructs on 8% urea PAGE. Synthesized constructs were used as ladders on both sides of the gel. Full blots are presented in Fig S7b and Fig S7c. (c) and (e) Reporter gene assays. *MAOA* promoter constructs carrying the 2R, 2.5R, 3R, 3.5R, 4R, 4.5R, 5R, 5.5R, or 6R allele fused to firefly luciferase were co-transfected with plasmid containing Renilla luciferase into SH-SY5Y cells and JAR cells. Empty pcDNA vector was used as a negative control. Data are presented as the ratio of firefly to Renilla luciferase (mean ± SEM) from one of two independent experiments with 3–4 wells. (d) and (f) represent the statistically significance for results of reporter gene assays in SH-SY5Y cells and JAR cells, respectively. * *p* < 0.05, ** *p* < 0.01, and *** *p* < 0.001 indicate statistically significant differences between 2-6R alleles and negative control group by one-way ANOVA with Tukey post hoc test.

**Supplementary Figure 2. Comparisons of self-report questionnaire scores between *MAOA* genotype.**

(a) and (b) represent total scores for the three questionnaires on aggression; (c) and (d) represent BPAQ subcategory scores; (e) and (f) represent PCS subcategory scores between *MAOA* genotypes in men (3.5R/Y vs. 4.5R/Y) and in women (3.5R/3.5R vs. 4.5R/4.5R vs heterozygous group), respectively. All values are presented as means ± SEM. Sample sizes are 334 for all questionnaires, 312 for BDHI, 312 for BPAQ, and 327 for PCS (BDHI, Buss-Durkee Hostility Inventory; BPAQ, Buss-Perry Aggression Questionnaire; PCS, Peer Conflict Scale). **p* < 0.05 indicates statistically significant differences between *MAOA* genotype in total scores of three self-reports by two-way ANOVA with Bonferroni post hoc test; **p* < 0.05 indicates statistically significant differences between *MAOA* genotype in subcategory scores of BPAQ and PCS by two-way ANOVA with Bonferroni post hoc test.

**Supplementary Figure 3. Comparison of EEG at F8 across *MAOA* genotypes.**

(a), (f), (k), and (p) represent the power spectral density of the α, β, θ, and δ waves at F8, respectively. The MSE values are for the following waves: (b) and (c) for α waves; (g) and (h) for β waves; (l) and (m) for θ waves; (q) and (r) for δ waves. These are the results during NS and S between *MAOA* genotypes in men and women. The relative power values are as follows: (d) and (e) for α waves; (i) and (j) for β waves; (n) and (o) for θ waves; (s) and (t) for δ waves. All values are presented as the means ± SEM. The EEG sample size was 84, including 36 men (21 for 3.5R/Y, 15 for 4.5R/Y) and 48 women, (23 for 3.5R/3.5R, 5 for 4.5R/4.5R, and 20 for the heterozygous group). * above the horizontal lines indicates statistically significant differences between *MAOA* genotypes at *p* < 0.05 by repeated measures of two-way ANOVA with the Bonferroni post hoc test; * above the graph bars indicates statistically significant differences between NS and S at *p* < 0.05 by repeated measures of two-way ANOVA with the Bonferroni post hoc test.

**Supplementary Figure 4. Comparison of EEG at Fp2 across *MAOA* genotypes.**

(a), (f), (k), (p), and (u) represent the power spectral density of the α, β, θ, δ, and γ waves at Fp2, respectively. The MSE values are for the following waves: (b) and (c) for α waves; (g) and (h) for β waves; (l) and (m) for θ waves; (q) and (r) for δ waves; and (v) and (w) for γ waves. These are the results during NS and S between *MAOA* genotypes in men and women. The relative power values, which are the mean value of the power spectral density at each frequency interval, are as follows: (d) and (e) for α waves; (i) and (j) for β waves; (n) and (o) for θ waves; (s) and (t) for δ waves; and (x) and (y) for γ waves. All values are presented as the means ± SEM. The EEG sample size was 84, including 36 men (21 for 3.5R/Y, 15 for 4.5R/Y) and 48 women, (23 for 3.5R/3.5R, 5 for 4.5R/4.5R, and 20 for the heterozygous group). * and ** above the horizontal lines indicate statistically significant differences between *MAOA* genotypes at *p* < 0.05 and *p* < 0.01, respectively, by repeated measures of two-way ANOVA with the Bonferroni post hoc test; * and ** above the graph bars indicate statistically significant differences between NS and S at *p* < 0.05 and 0.01, respectively, by repeated measures of two-way ANOVA with the Bonferroni post hoc test.

**Supplementary Figure 5. Time-line for stimuli in video.**

The video has a full length of 118 sec. After a fixation cross appeared, neutral scene (NS), scene 1 (S1), scene 2 (S2), and scene 3 (S3) were presented. Between-scene interval has a duration of 3-5 sec.

**Supplementary Figure 6. Comparison of EEGs in the Fp2, F8, P7, and P8 channels.**

(a), (b), and (c) represent the MSE values of the α, β, and γ waves during NS and S at four channels of Fp2, F8, P7, and P8, respectively. (d), (e), and (f) represent the relative power values of the α, β, and γ waves. All values are presented as the means ± SEM. The EEG sample size was 32, including 18 men and 14 women. * and ** above the graph bars indicate statistically significant differences between NS and S at p < 0.05 and 0.01, respectively, by the two-tailed independent *t-test*.

**Supplementary Figure 7. Full-length blots or gels of included figures.**

(a) Full-length agarose gels from Fig 1b. (b) and (c) Full-length urea PAGE from Fig S1a and Fig S1b, respectively. Lane I in each blot represents synthesized 2.5R, 3.5R, 4.5R, and 5.5R constructs, from bottom to top. Lane II in each blot represents synthesized 2R, 3R, 4R, 5R, and 6R constructs, from bottom to top.


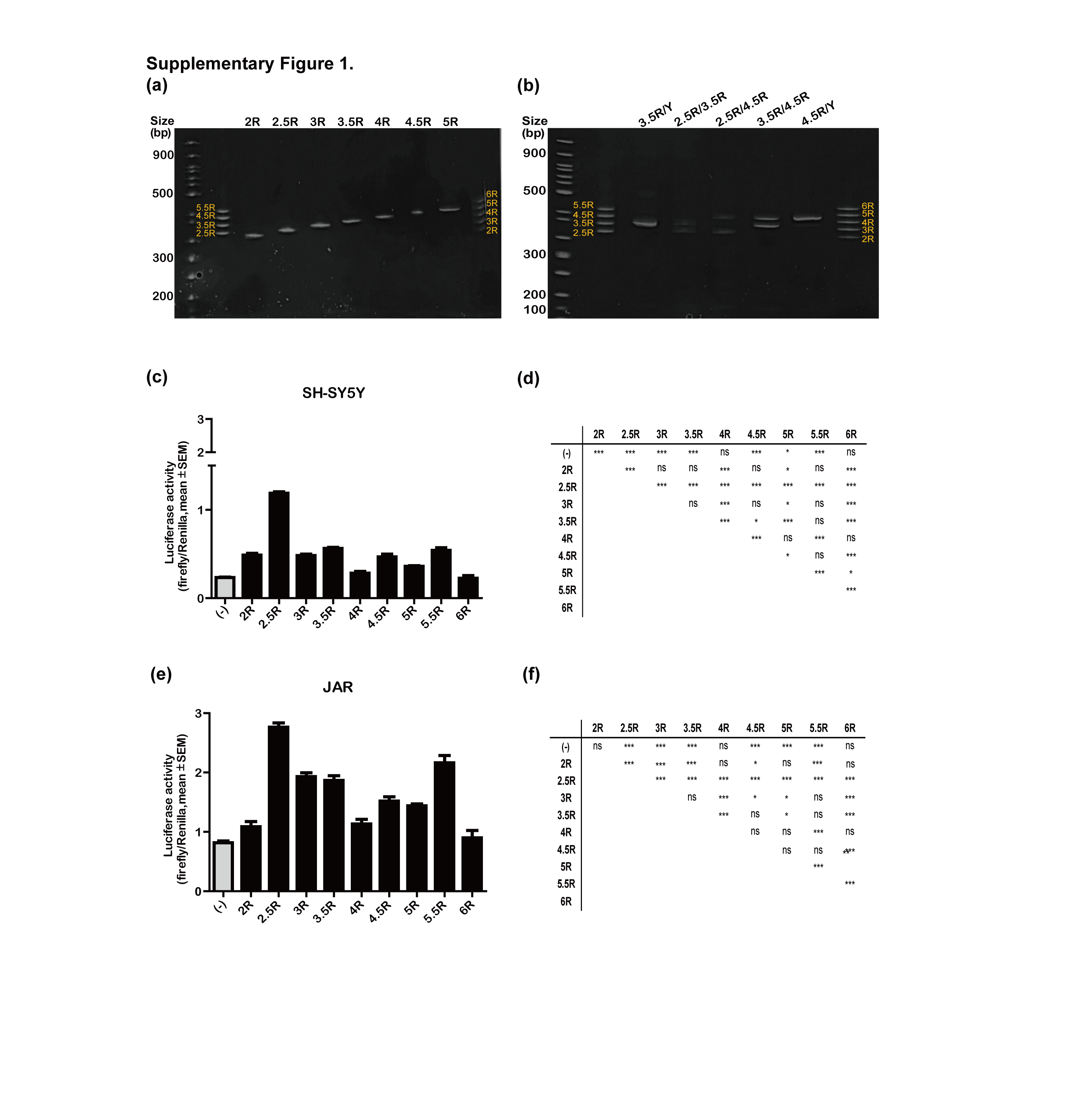


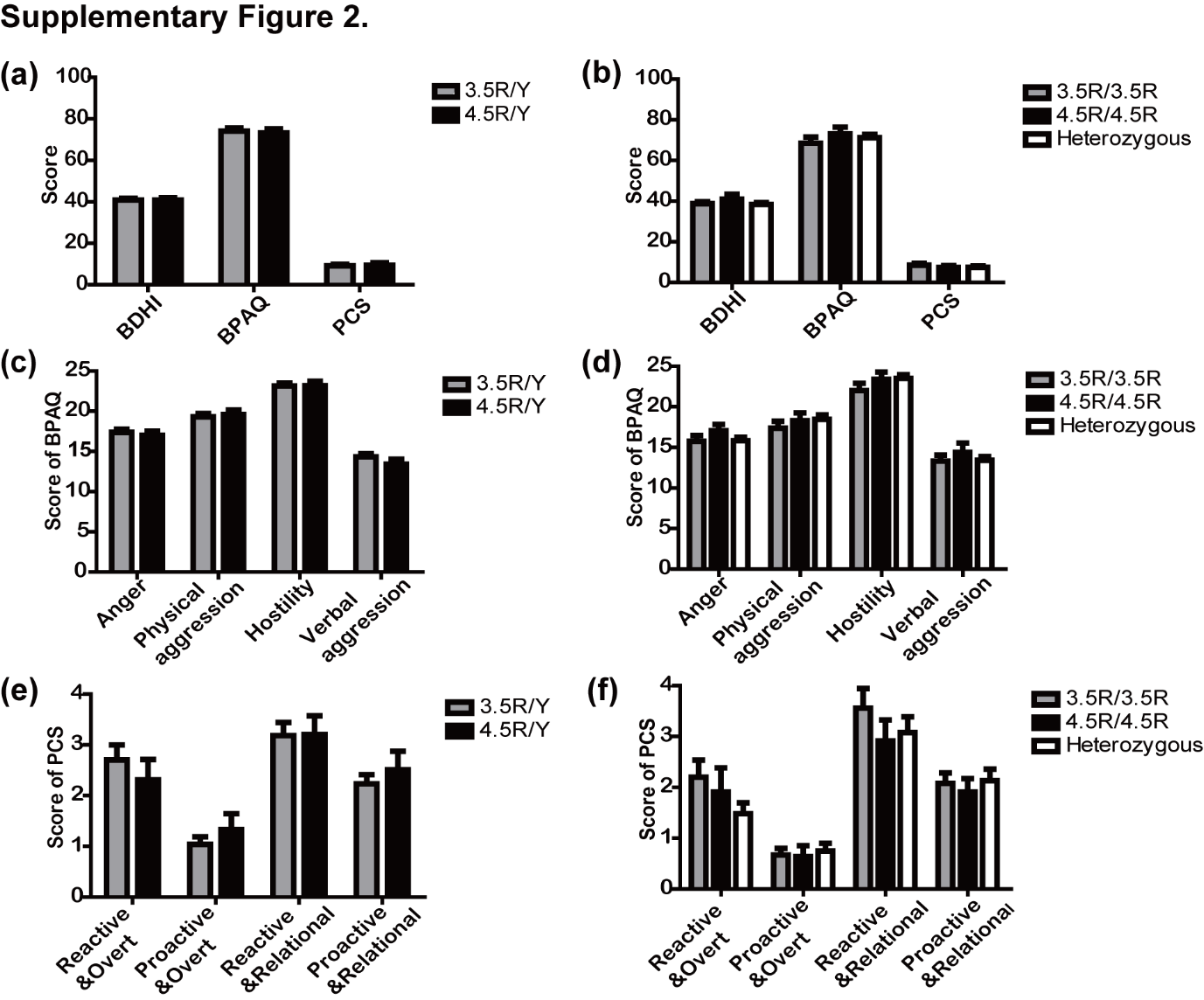


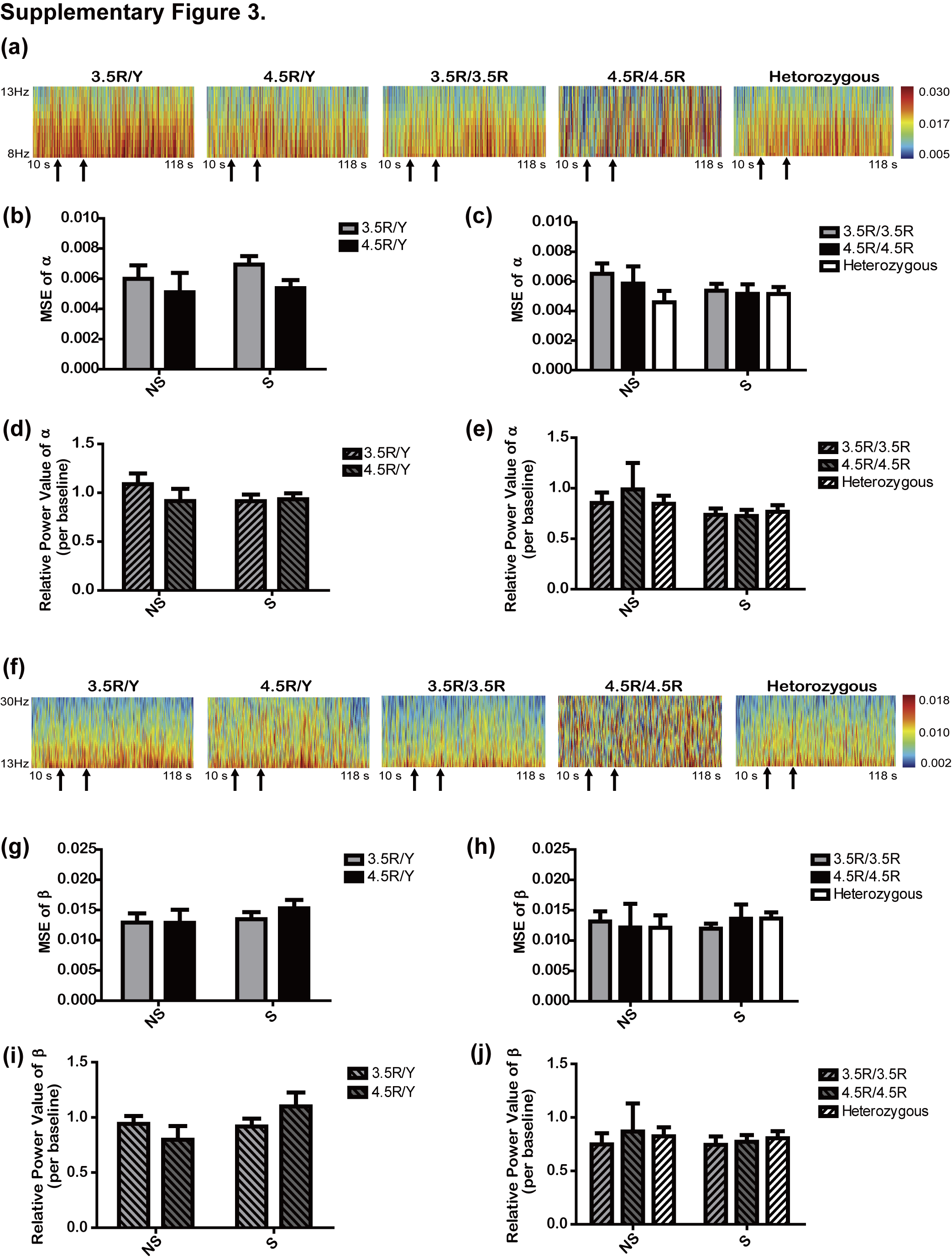


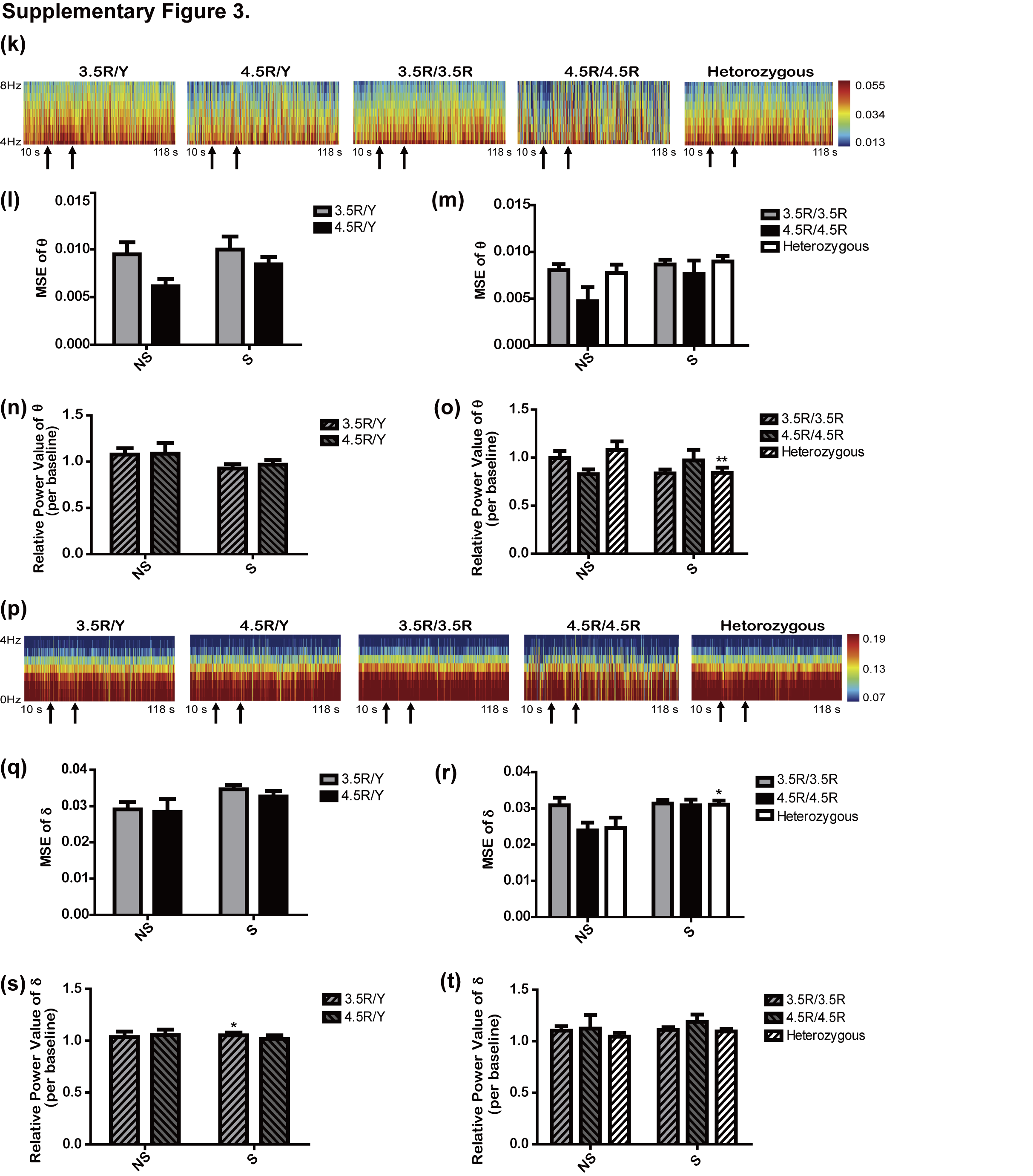


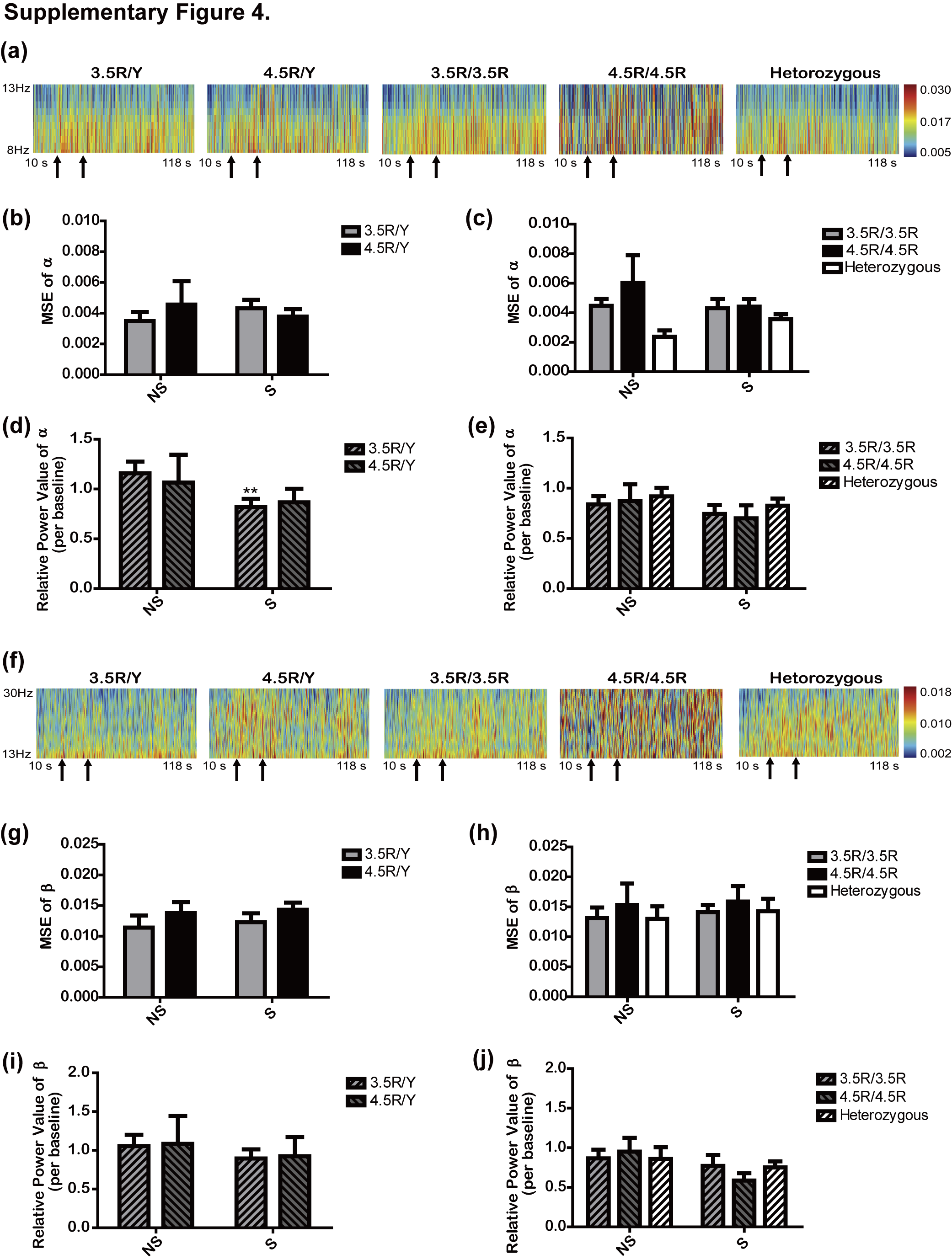


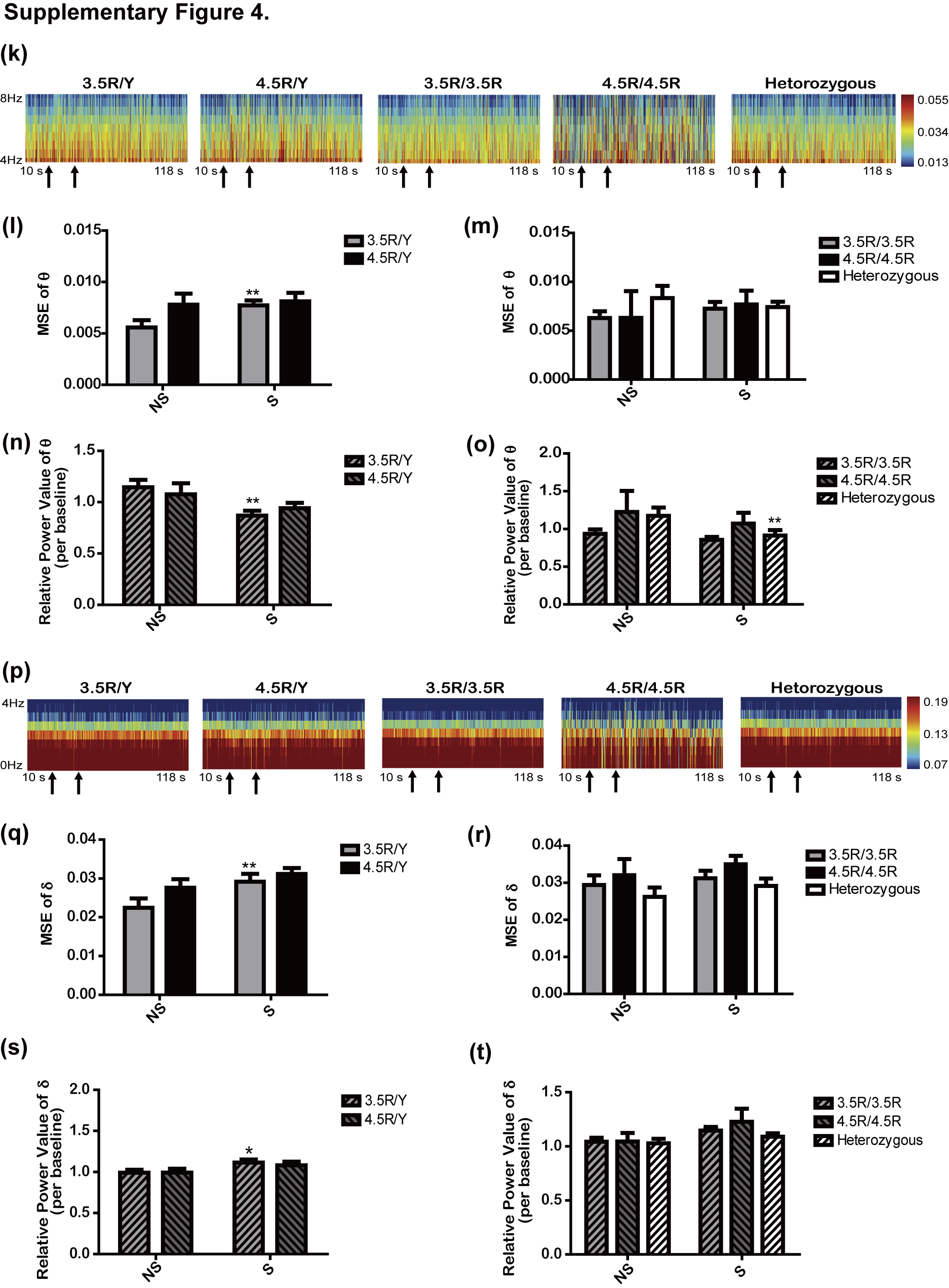


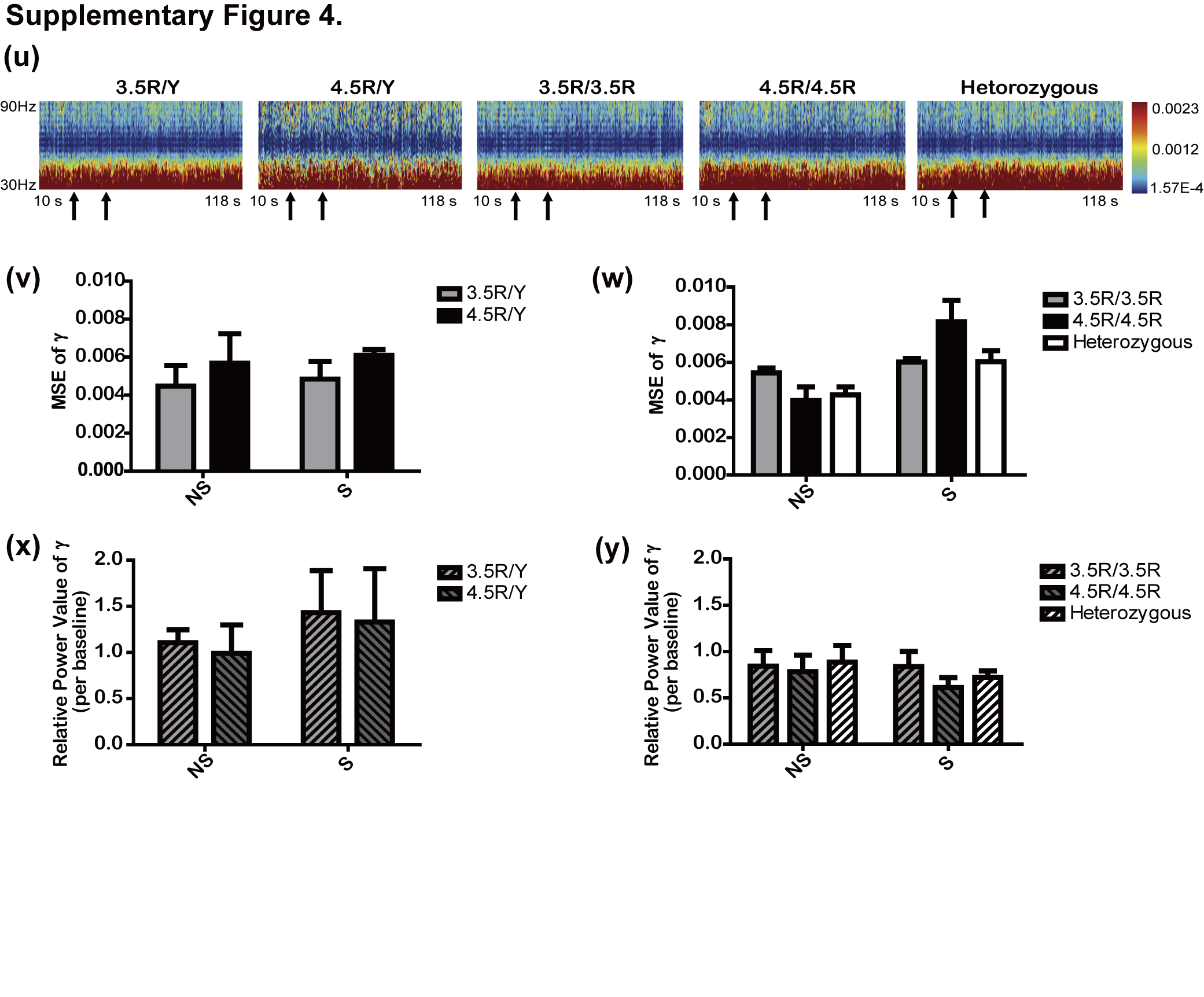


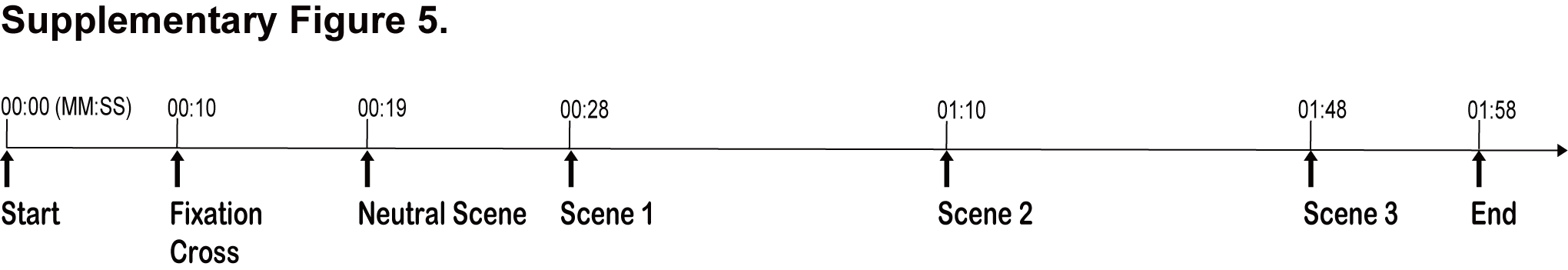


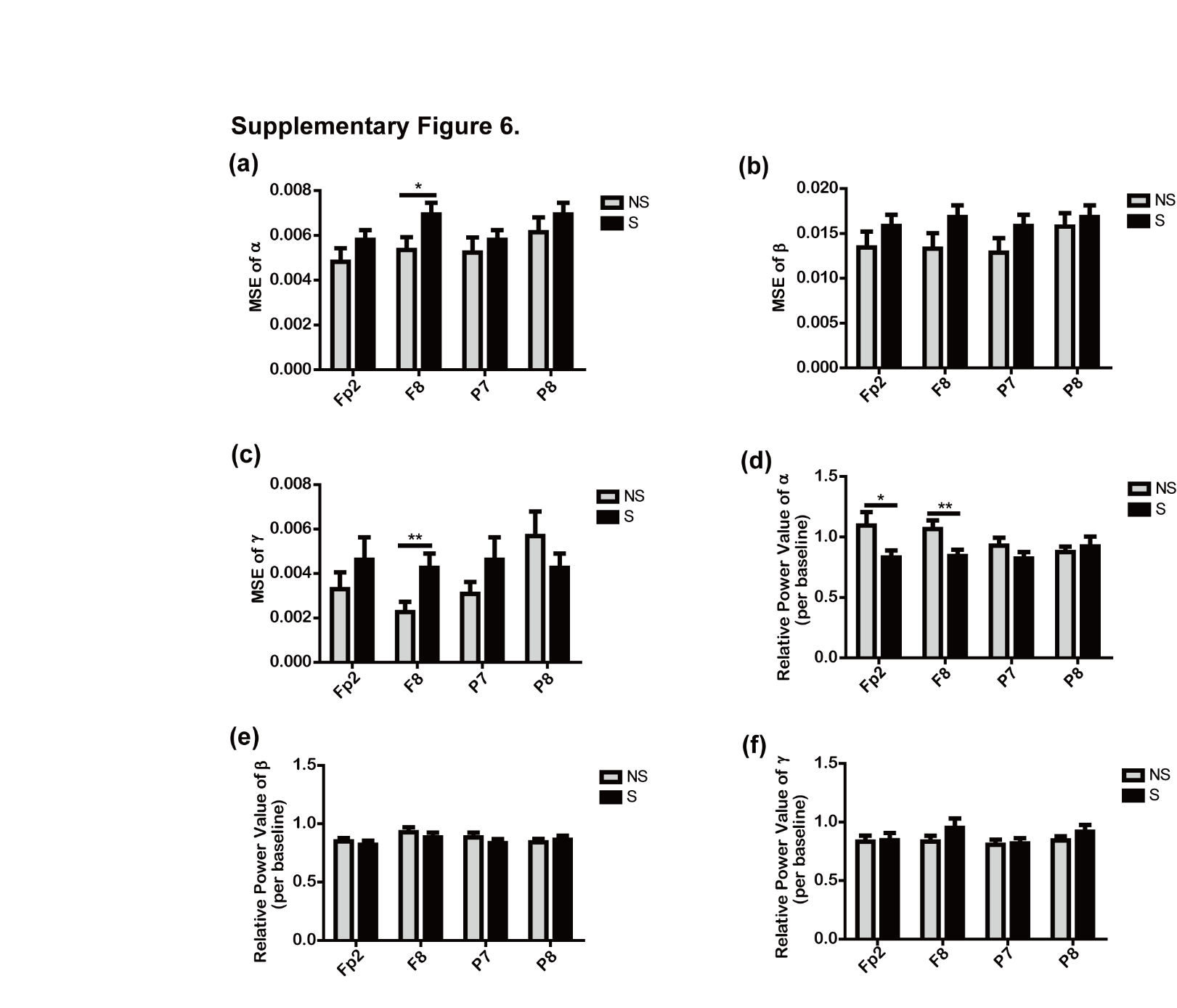


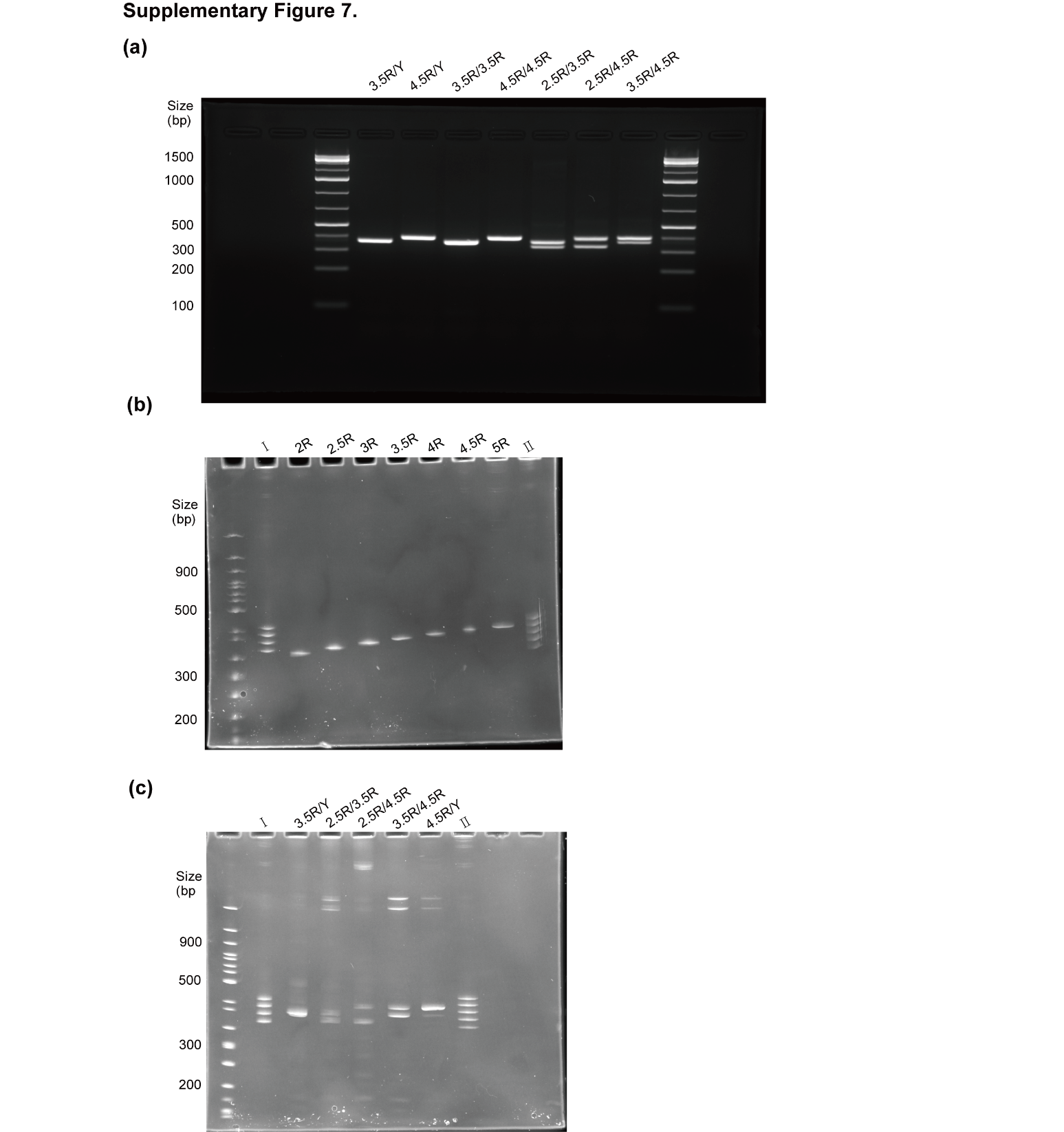

Supplement: Supplementary file 1 — Supplementary information [file 41598_2019_39103_MOESM1_ESM.doc]
